# Extent of positive surgical margins following radical prostatectomy: impact on biochemical recurrence with long-term follow-up

**DOI:** 10.1186/s12894-019-0470-8

**Published:** 2019-05-15

**Authors:** Yoann Koskas, François Lannes, Nicolas Branger, Sophie Giusiano, Nicolas Guibert, Géraldine Pignot, Jochen Walz, Dominique Rossi, Cyrille Bastide

**Affiliations:** 1 Department of Urology, Hopital Nord, Chemin des Bourrely, 13015 Marseille, France; 2 Department of Anatomic Pathology, Hôpital nord, Chemin des Bourrely, 13015 Marseille, France; 3 Centre hospitalier universitaire de Lyon, Lyon, France; 4 Departement of urology, Institut Paoli-Calmettes, 13008 Marseille, France; 5 Hopital Edouard Herriot, 69003 Lyon, France

**Keywords:** Prostate cancer, Radical prostatectomy, Biochemical recurrence, Positive surgical margins, Extent, Focal positive surgical margins

## Abstract

**Background:**

To assess the prognostic value of the extent of positive surgical margins (PSM) following radical prostatectomy (RP) on biochemical recurrence (BR) with long-term follow-up.

**Methods:**

This retrospective study analyzed 1275 RPs performed between January 1992 and December 2013 in two university centers in Marseille (France). The inclusion criteria were: follow-up > 24 months, undetectable postoperative prostate-specific antigen (PSA), no seminal vesicle (SV) invasion, no lymph node invasion confirmed by surgery (pN0) or imaging (pNx), and no neoadjuvant or adjuvant treatment. BR was defined by PSA level ≥ 0.2 ng/mL on two successive samples. We included 189 patients, divided into two groups:

- Focal PSM (fPSM): single PSM (sPSM) ≤3 mm;

- Extensive PSM (ePSM): sPSM with linear length > 3 mm or several margins regardless of the length.

**Results:**

The median follow-up was 101 months (18–283) and the median age was 63 years (46–76). BR occurred in only 12.1% (14/115) of cases involving fPSM and in 54.1% (40/74) of cases involving ePSM. In the multivariate model, ePSM patients were significantly associated with increased BR compared to fPSM (hazard ratio [HR] = 6.11; 95% confidence interval [CI] = 3.25–11.49). The ePSM significantly decreased BR-free survival (*p* < 0.001) for every patient and every subgroup (pT2, pT3a, pG ≤6, and pG ≥7). The median BR time following RP was significantly shorter for ePSM patients than fPSM (57.2 vs. 89.2 months *p* < 0.001).

**Conclusion:**

With a median 8-year follow-up, ePSM was strongly associated with BR compared to fPSM. Therefore, it seems legitimate to monitor patients with fPSM. In cases of ePSM, adjuvant treatment appears effective.

## Background

The presence of positive surgical margins (PSM) following radical prostatectomy (RP) is a worrying for surgeons and patients alike as it represents an independent biochemical recurrence (BR) risk factor [1–8]. The rate of PSM is 5–30% for organ-confined prostate cancer and 17–65% for locally-advanced cancer [9–11].

A PSM means incomplete cancer resection and may lead to additional treatment, such as either adjuvant radiotherapy (AR) or chemical or surgical castration. These remedial treatments display side effects and affect patient quality of life [12]. However, despite the evidence that AR significantly reduces the BR risk in locally-advanced cancers [12], the optimal approach concerning PSM remains unclear. The strategy eventually employed varies according to institution, while often left to the surgeon’s discretion or simply following each team’s habitual practice.

Moreover, there is generally no evaluation of PSM characteristics (size, number, location, and focality) regarding these additional therapeutic decisions. This can be explained by the observation that there is no consensus yet taking into account these factors for the therapeutic decision.

One reason could be the lack of standardization of anatomopathological reports regarding PSM description, and thus a potential lack of information that could be detrimental to the therapeutic decision-making process. The College of American Pathologists recommends referring to the Gleason score of the margin [13].

Nevertheless, there are still no clear recommendations, especially regarding how to act depending on the PSM (length and number) extent. Lake et al. [14] reported that the extent of the margin significantly affects BR-free survival, which is improved in cases of fPSM (length ≤ 3 mm) compared to ePSM (multifocal PSM or length > 3 mm).

In another recent study based on a median 52-month follow-up, Lee et al. [15] demonstrated that ePSM can significantly affect BR-free survival compared to fPSM. It should, however, be noted that their study included patients classified N+ and/or pT3b, which could be confounding factors in the BR assessment. Similarly, Maxeiner et al. [16] reported that multiple PSM and those > 3 mm represented an independent BR factor, whereas these authors likewise included patients with potential confounding factors (N+, PT3b, PT4, or neoadjuvant treatment).

In our study involving a median 5-year follow-up, we sought to assess the BR risk in relation with PSM extent following RP for Stage ≤PT3a patients who had not received any neoadjuvant or adjuvant treatment. We also sought to determine the influence of other BR prognostic factors, such as PSM location within the prostate.

## Methods

From January 1992 to December 2013, a total of 1275 patients underwent RP for prostate cancer in two university centers in Marseille (France). Using a prospective database, we retrospectively included patients exhibiting PSM who met the following criteria: follow-up > 18 months, undetectable postoperative PSA, no invasion of the SV, absence of lymph node invasion confirmed by surgery (pN0) or imaging (pNx), and no neoadjuvant or adjuvant treatment. The surgical techniques for RP differed among cases: open RP, laparoscopic RP (LP-RP), and robot-assisted laparoscopy (RALP-RP). Each intervention was conducted by experienced surgeons.

BR was defined as PSA level ≥ 0.2 ng/mL on two successive samples. The anatomopathological examination of all surgical specimens was performed according to the Stanford technique [17] up to 2009, and then as recommended by the ISUP from 2009 onwards [3]. A centralized reading of the slides was performed by the same pathologist. Positive margins were defined as the presence of cancerous tissue in contact with the inked surface of the prostatectomy specimen. Healthy tissue margins were considered negative margins.

The extent of the margin was assessed as follows:

- fPSM: single margin with a linear length ≤ 3 mm present on one cutaway view;
- ePSM: single margin with a linear length > 3 mm present on one cutaway view, single margin (regardless of the length) present on several cutaway views, or multiple margins present on one or more cutaway views.

For each patient, the location of the PSM *vis-a-vis* the prostate was likewise recorded and classified as follows: bladder neck PSM, basis PSM, posterolateral PSM, anterior PSM, and apex PSM. Additionally, the weight and volume of the prostate were listed.

We were then able to evaluate BR-free survival and BR risk depending on the PSM extent and search for other BR risk factors, such as the preoperative PSA level, Gleason score on the specimen, tumor stage (pT), and PSM location.

All statistical analyses were performed using the statistical software R Version 2.5.3. The statistical tests were bilateral and *p*-values ≤0.05 were considered significant. The comparisons of percentages were performed using the Chi-squared test, comparisons of means using Student’s t-test, and comparisons of survival using either the LogRank test for bivariate situations or the Cox model for multivariate situations. Survival curves were plotted according to the Kaplan-Meier method.

## Results

Overall, 189 patients exhibiting PSM who met the inclusion criteria were included in the study. The median follow-up was 101 months, while median age and preoperative PSA were 63 and 7.7 ng/mL, respectively. In total, 25% of patients were pT3a, and the remainder in pT2. Only 7% had a Gleason score ≥ 8, the majority (63%) were pG = 7. According to the D’Amico classification, 55% were low-risk, 34.4% intermediate-risk, and only 10.6% high-risk.

No differences regarding Gleason score, pT stage, or surgical technique were observed between the two subgroups. Lymph node dissection was performed on 117 patients (61.9%), whereas the others were classified as cN0 based on imaging.

The most often-performed RP technique was open RP (127 patients, 67.7%), with RALP RP performed on 61 patients (32.3%), and LP RP on only one case. The clinical and histological patient characteristics patients have been summarized in Table 1.

**Table 1:** Clinical and histological characteristics of patients according to the extent of positive surgical margin (PSM)

	Total cohort n (%)	fPSM n (%)	ePSM n (%)	p
Patients (n)	189	115 (60.9)	74 (39.1)	
Mean age (standard deviation)	71.8 (+/−6.3)	70.3 (+/−7.5)	74.1 (+/−8.3)	0.006
Mean PSA (standard deviation)	8.8 (+/−4.3)	8.1 (+/−3.6)	10.0 (+/−4.9)	0.003
Gleason Score (pG)				0.14
≤ 6	63 (33.3)	43 (37.4)	20 (27)	
7	119 (63)	68 (59.1)	51 (68.9)	
3 + 4	96 (49.2)	57 (49.6)	36 (48.6)	
4 + 3	23 (13.8)	11 (9.6)	15 (20.3)	
≥8	7 (3.7)	4 (3.5)	3 (4)	
pT Stage				0.61
T2a	15 (7.9)	9 (7.8)	6 (8.1)	
T2b	24 (12.7)	16 (13.9)	8 (10.8)	
T2c	101 (53.4)	64 (55.6)	37 (50)	
T3a	49 (25.9)	26 (22.6)	23 (31.1)	
Median follow-up (months)	101.1	95.3	110.3	0.08

*fPSM* focal positive surgical margin, *ePSM* extensive positive surgical margin, *PSA* prostate-specific antigen

BR was observed in only 54 patients (28.6%). In the fPSM group, 14 (12.2%) suffered from recurrence, compared to 40 (54%) in the ePSM group. The clinical and histological features of these patients have been detailed in Table 2.

**Table 2:** Clinical and histological characteristics of patients exhibiting biochemical recurrence (BR)

	Total PSM	fPSM	ePSM	p
N	54	14	40	
Mean PSA	9.0	6.4	9.5	0.001
pT Stage				
pT2 n (%)	31 (57.4)	9 (64.3)	22 (55.0)	0.081
pT3a n (%)	23 (42.6)	5 (35.7)	18 (45.0)	0.010
Gleason score pG				
pG ≤ 6 n (%)	17 (31.5)	2 (14.3)	15 (37.5)	0.002
pG = 3 + 4 n (%)	19 (35.2)	6 (42.9)	13 (32.5)	0.023
pG = 4 + 3 n (%)	13 (24.0)	4 (28.6)	9 (22.5)	0.097
pG ≥ 8 n (%)	5 (9.3)	2 (14.2)	3 (7.5)	0.174

*fPSM* focal positive surgical margin, *ePSM* extensive positive surgical margin, *PSA* prostate-specific antigen

**Fig. 1:**
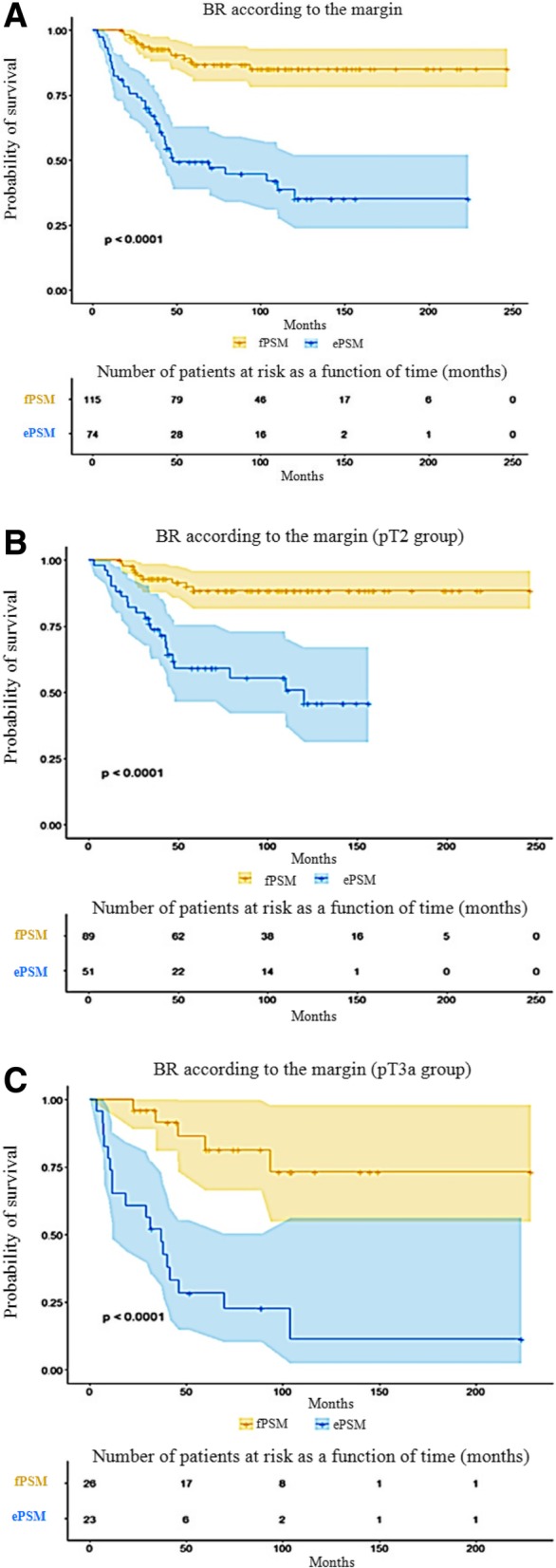
Kaplan-Meier curves showing biochemical recurrence (BR)-free survival following. **a** All patients. **b** pT2 Stage. **c** pT3a Stage. Legends: fPSM: focal positive surgical margin; ePSM: extensive positive surgical margin

BR-free survival at 5 years was 86.8% for patients with fPSM versus 49.4% for those with ePSM, and at 8 years 85.1 and 44.8%, respectively (Fig. 1). Patients with ePSM were significantly more likely to develop BR (*p* < 0.0001), regardless of the subgroup. This significant relationship was found even for patients classified as pT3a or pG ≥7, albeit less pronounced.

In the univariate and multivariate models (Table 3), ePSM was strongly and significantly associated with BR, compared to fPSM (HR = 6.11; 95% CI = 3.25–11.49 on multivariate analysis). There was likewise a link between pT stage and BR occurrence: pT3a stages were associated with a higher risk of recurrence compared to pT2 stages (HR = 2.14; 95% CI = 1.20–3.81 on multivariate analysis). The preoperative PSA > 10 was not significantly associated with BR (HR = 1.33; 95% CI = 0.74–2.39) as well as Gleason score (pG > 6) on multivariate analysis. On univariate analysis, pG = 4 + 3 and pG ≥8 was significantly associated with BR (2.54 [1.23, 5.23] and 2.77 [1.02, 7.52], respectively).

**Table 3:** Univariate and multivariate model and hazard ratio calculations for variables associated with biochemical recurrence

Variables	Univariate		Multivariate	
PSA (PSA > 10 vs ≤ 10)	1.45 (0.82–2.58)	0.205	1.33 (0.74–2.39)	0.339
pT (pT3a vs pT2)	2.58 (1.5–4.44)	< 0.001	2.14 (1.2–3.81)	0.01
pG ≤6	Reference		Reference	
pG = 3 + 4	NS		NS	
pG = 4 + 3	2.54 (1.23–5.23)	0.012	1.15 (0.53–2.47)	0.724
pG ≥8	2.77 (1.02–7.52)	0.046	1.41 (0.5–3.99)	0.513
PSM (ePSM vs fPSM)	6.05 (3.29–11.15)	< 0.001	6.11 (3.25–11.49)	< 0.001

The multivariate model includes all variables in this table, the age when the RP was performed and the year of treatment

*HR* hazard ratio, *CI* confidence interval, *fPSM* focal positive surgical margin, *ePSM* extensive positive surgical margin, *PSA* prostate-specific antigen

In the multivariate model, an interaction between Gleason score (pG) and the PSM extent was tested, as well as between pT stage and the PSM extent. In both cases, the correlations were found to be not significantly different from 1.This observation suggests that the effect of the PSM extent on BR does not depend on the Gleason score or pT stage.

Concerning the loco-regional and remote evolution of the disease, 1/189 patients with fPSM became metastatic 16 years following RP, while 1 patient became lymph-node metastatic after 6 years.

Median BR time was significantly shorter for patients with ePSM compared to those with fPSM: 89.2 months in the fPSM group versus 57.2 months in the ePSM group.

Regarding the PSM characteris, the most common location was apical, exhibited in 99 patients (53%) The posterolateral location was the second most common, involving 79 patients (42%). For the basal PSM, 37 patients (20%) exhibited this location associated or not with another. Anterior and bladder-neck PSMs were significantly less frequent, with 1.5% exhibiting bladder-neck PSM and 2% anterior PSM. Multiple PSMs significantly decreased BR-free survival compared to single PSM locations.

No single PSMs significantly affected BR-free survival, although basal PSMs tended to decrease BR-free survival compared to the apical and posterolateral PSMs [22-24]

## Discussion

To our knowledge, this is the first study with a median follow-up of 8 years that found no association between fPSM and BR in patients who had received no neoadjuvant or adjuvant treatment and exhibited slightly locally-advanced disease (<pT3b and N0).

This long-term data taken from a selected population enabled us to evaluate the PSM on BR. In this multicenter study with an 8-year median follow-up, we demonstrated that men with ePSM following prostatectomy were significantly more likely to develop BR than those with fPSM. Additionnally, the more extended the PSM, the shorter the BR time.

Other studies in the literature specifically analyzed the prognostic value of the PSM extent following prostatectomy for patients followed-up for prostate cancer.

Sooriakumaran et al. [18] analyzed a large series of 189 patients with PSM involving a minimum follow-up of 5 years. These authors reported that patients with PSM > 3 mm had significantly higher risk of developing BR. This study excluded patients who had neoadjuvant or adjuvant treatment, yet did include pT3b patients. This a potential confounding factor. For this reason we have deliberately excluded all potential confounders of RB.

Therefore, we intentionally excluded patients with locally-advanced disease (lymph node or seminal vesicle invasion) in order to better select patients whose BR was most likely to be linked to PSM. For patients with locally-advanced disease, BR is most likely a reflection of micro-metastatic systemic disease, meaning the PSM status in these cases would have a very limited influence on BR [19, 20].

An analysis of a large cohort identifying 498 patients with PSM reinforced this hypothesis [21], demonstrating in multivariate analysis that PSM was an independent factor of BR for patients classified as pT2 and pT3a (*p* < 0.001; HR = 3.81; *p* = 0.001; HR = 2.09, respectively), whereas this was not so for pT3b and pT4 patients (*p* = 0.196 and *p* = 0.061, respectively).

All in all, for patients with lymph node or seminal vesicle invasion, the prognosis does not really seem related to PSM changes, but rather to the existence of micro-metastases responsible for the systemic spread of the disease. This is why we excluded these patients from our study.

Moreover, even if this hypothesis proved inaccurate, the postoperative measures would be the same. With or without PSM, it is still recommended to perform adjuvant therapy in patients classified as pT3b and pT4 [12]. It is therefore of little interest to focus on patients with locally-advanced disease in a study of PSM, as long as the results do not impact or only slightly impact the measures to be taken.

Regarding the length of the PSM, other studies have analyzed the effect, though often their populations were not selected to eliminate potential confounder BR factors.

Nevertheless, in large series of 501 patients, Stephenson [26] showed that the number of PSM was a significant predictor of BR, with multiple PSM resulting in worse prognosis compared to a solitary PSM (adjusted HR = 1.4, 95% CI: 1.1–1.8, *p* = 0.002). In parallel to this, ePSM was associated with BR, versus fPSM (adjusted HR = 1.3, 95% CI: 1.1–1.6, *p* = 0.004).

BR free-survival was significantly worse for patients with ePSM than those with fPSM (49.4 and 86.8%, respectively at 5 years, *p* < 0.0001) in our study. This result is widely found in other studies (Table 4).

**Table 4:** Hazard ratios of biochemical recurrence (BR) in multivariate model and BR-free survival following radical prostatectomy in men with fPSM or ePSM

Study	Year	n	fPSM no. (%)	ePSM no. (%)	Median follow-up, yr.	HR for BR (95% CI) ePSM vs fPSM *p* value	BR-free survival at 5 years for fPSM (%)	BR-free survival at 5 years for ePSM (%)	Including ≥pT3b, N+, neo or adjuvant treatment
Lake et al. [14]	2010	2022	344 (17.0)	99 (4.9)	4.1	NR	72	62	Yes
Lee e al [15]	2015	1733	114 (6.6)	359 (20.7)	4	NR	83	54	Yes
Sooriakumaran et al. [25]	2013	893	100 (11.2)	81 (9.1)	5	2.43 (1.14–5.18) < 0.05	81	65	Yes
Stenphenson et al. [27]	2009	1501	983 (65.5)	518 (34.5)	7	1.3 (1.1–1.6) *0.004*	63	43	No
May et al. [28]	2011	1036	122 (11.8)	145 (14.0)	4.3	1.0 (0.66–1.55) 0.96	49	51	No
Porpiglia et al. [29] ^a^	2012	300	48 (16.0)	20 (6.7)	5.1	5.7 (1.5–2.17) 0.01	77	55	Yes
Ochiai et al. [30]	2007	117	81 (69.2)	32 (27.4)	3.6	NR	84	52	Yes
Van Oort et al. [31]	2010	174	NR	NR	3.6	2.15 (1.12–4.2) 0.02	NR	NR	Yes

In all studies, the cut-off for fPSM was the same as in our study (single margin ≤ 3 mm), except for Porpiglia et al.^a^ (≤2.8 mm)

*NR* not reported, *HR* hazard ratio, *CI* confidence interval, *fPSM* focal positive surgical margin, *ePSM* extensive positive surgical margin, *PSA* prostate-specific antigen

One limitation of our study was the retrospective nature of our analysis.

In addition, while the number of patients analyzed was lower than that of some studies, we voluntarily restricted the study population (N +, ≥ T3b, adjuvant or neoadjuvant excluded) in order to get a selected population that could clearly reflect the effects of PCM. Only very few authors have investigated a population equally absent of BR confounders.

Lastly, we did not adjust our results according to tumor volume and the surgeon’s experience, though these two variables constitute established risk factors for BR [25].

Also, in terms of limits, the Gleason score of the PSM could have been analyzed in order to reveal any possible link with BR. However, within the Marseille centers involved in our study, the Gleason score is either barely or not at all analyzed on PCMs.

Nevertheless, in agreement with a recent review, the impact of the Gleason score of PSM on BR is not currently acknowledged since too few studies have analyzed this factor [10]. We need better accuracy in analysis of PSM by pathologists if we are to move forward on this issue.

Regarding the primary endpoint, we choose the BR. This criterion is the most widely used in the literature for this type of analysis and certainly a relevant criterion when it comes to assessing disease recurrence. Nevertheless, the consideration of other criteria, such as metastasis-free survival, resistance to castration, or specific mortality to prostate cancer, would also be of value. In our study, only one patient (0.5%) developed metastatic recurrence after 16 years of follow-up, and no patients died from prostate cancer.

In a recent study, Mauermann investigated these factors in a cohort of 1712 patients, including 591 patients with PSM [8]. Patients with PSM were divided into two groups: single margin (regardless of size) and multiple margins. In total, 1.3% became metastatic, 1% became resistant to castration, and 1% died of prostate cancer. In the multivariate model, PSM (single or multiple) were never significantly associated with metastatic disease, resistance to castration, or specific mortality. In contrast, PSM (single and multiple) were a risk factor for BR. Again, this study included patients classified as pT3b, pT4, and N +, which likely accounts for this lack of significance.

Finally, the question remains whether treatment deferral should be recommended only when BR occurs. This can prevent a number of patient side effects, for example in our study, 49.4% of patients with ePSM exhibited no BR with a median follow-up of 8 years.

Three large randomized trials compared AR vs. wait and see policy following prostatectomy for high-risk prostate cancer. The European Organization for Research and Treatment of Cancer (EORTC 22,911) and the German Cancer Society (ARO 96–02) trial (31, 32) reported a significantly improved 10-year cumulative BR-free survival for the postoperative irradiation group vs. the observation group. Overall survival and clinical progression were not significantly affected. The Southwest Oncology Group (SWOG) 8794 trial reported greater metastasis-free survival and overall survival in the postoperative radiotherapy arm (33). It should be note that in these three trials, patients in the wait and see arm who presented with BR did not undergo radiotherapy early on (early salvage radiation therapy), meaning this treatment was less effective due to being performed late.

Also, Briganti [25] studied this very point in a series of 390 patients. His objective was to evaluate BR-free survival in patients receiving AR versus observation only, followed by early salvage radiotherapy in cases of relapse in patients undergoing RP for pT3, pN0, and R0–R1 disease. There was no difference found between the two groups. It is important to note that the margin was not taken into account in this analysis, however.

A French study is currently in progress (GETUG/AFU 17) that could perhaps give more precise results in this matter.

In the end, the therapeutic dilemma for patients with PSMs following radical prostatectomy is to distinguish those who need adjuvant therapy from those for whom simple monitoring would suffice. While it is still unclear what the best treatment is for patients with PSMs, our data may provide beneficial information regarding how to best proceed, particularly for patients with fPSM.

## Conclusion

Our study strongly suggests that the PSM extent should be taken into account in therapeutic decisions following radical prostatectomy. The existence of fPSM does not constitute a poor prognosis factor, as it was very rarely found associated with BR in our study. Therefore, it seems legitimate for us to propose close monitoring in these cases.

For patients with ePSM, however, the question remains whether early treatment would be beneficial. Our study clearly indicates that these patients much more frequently suffer BR, with 45% recurrence at 8 years, and it would thus appear essential to treat them early. Nevertheless, in these cases we believe it essential that the final therapeutic decision integrate the other prognostic factors (Gleason, preoperative PSA, pT) and life expectancy in order to treat these patients as well as possible. Further studies are now required to determine whether early salvage RT or RT associated with chemical castration are an equivalent alternative.

## Abbreviations

AR: Adjuvant radiotherapy
BR: Biochemical recurrence
ePSM: Extensive positive surgical margin
fPSM: Focal positive surgical margin
LP-RP: Laparoscopic radical prostatectomy
PSA: Prostate-specific antigen
PSM: Positive surgical margin
RALP-RP: Robot-assisted laparoscopy radical prostatectomy
RP: Radical prostatectomy
sPSM: Single positive surgical margin
SV: Seminal vesicle
